# From astronauts to stroke survivors: how the TheraSuit Method® can boost balance and recovery

**DOI:** 10.3389/fneur.2025.1581256

**Published:** 2025-08-13

**Authors:** Rose Lampert, Rahul Goel, João V. Oblanca, Daniel F. Martins

**Affiliations:** ^1^Experimental Neuroscience Laboratory (LaNEX), University of Southern Santa Catarina (UniSul), Palhoça, Brazil; ^2^Postgraduate Program in Health Sciences, University of Southern Santa Catarina (UniSul), Palhoça, Brazil; ^3^Independent Biomedical Researcher, Houston, TX, United States; ^4^Department of Physical Education, Maringa State University, Maringa, Brazil

**Keywords:** stroke, proprioception, rehabilitation, microgravity, bed rest, TheraSuit Method®, axial-loading suit

## Abstract

The human body exhibits remarkable adaptability to diverse environments. Astronauts in microgravity experience physiological changes like those observed in stroke patients due to inactivity. This shared challenge inspires the exploration of rehabilitation methods, bridging the gap between space medicine and physical therapy. This perspective examines the physiological similarities between microgravity and stroke, focusing on proprioceptive deficits. We then introduce the axial loading suit astronauts use to counteract these deficits and its potential application in stroke rehabilitation. We propose the TheraSuit Method®, a suit utilizing similar principles, as a promising tool to enhance whole-body proprioception and facilitate muscle activation against gravity, thereby promoting strength and functional recovery in stroke patients.

## Introduction

One of the brain’s primary functions is to receive sensory input from the environment and produce an appropriate motor response, i.e., body movement. The brain depends on blood flowing freely to deliver oxygen to the different areas to function correctly. A vascular interruption, like a stroke, can compromise parts of the brain that can get damaged without oxygen and lose important function (1). Every year, it is estimated that nearly 800,000 people are affected by a stroke in the United States (2), i.e., one roughly every 40 s.

Strokes disrupt communication between neurons (3), and can present with a wide range of symptoms, varying from altered cognition, paresis, muscle atrophy, absence of movement, vestibular disturbances, and difficulty standing and maintaining balance. This reduces overall independence and function and deteriorates the quality of life (4, 5).

Rehabilitation is a necessary intervention post-stroke to improve strength, balance and gait, and diminish sedentarism that can occur after hospitalization (4, 6). Different rehabilitation strategies have been applied to facilitate healing, such as mobilization, resistive exercises, neuromuscular electric stimulation, transfers from bed to sitting on a chair, and ambulation when possible (7–9). However, there is a lack of consensus on universally established rehabilitative strategies, including frequency and duration (10). During the first few weeks post stroke, an intensive intervention (i.e., carried out daily for at least an hour) may be applied to preserve motor maps and take advantage of enhanced neuroplasticity (10), which could increase sensory responses and encourage motor engagement (11). Maintaining strength can help preserve muscle mass, additionally helping speed recovery (12).

Stroke causes impairments of proprioception that translate into an altered sensory system (13). Proprioception is part of the sensory system that is necessary for premotor activation. Proprioception is codependent on muscle contraction to send signals to the receptors in the joints, Golgi tendon organs, and muscle spindles to activate somatic responses to adjust muscle tone (13). One common consequence of impaired proprioception after a stroke is the neglect of the affected side of the body. This neglect can manifest in various ways, with one notable impact on weight-bearing activities. Individuals may unintentionally favor their unaffected side, leading to an uneven weight distribution during activities like standing or walking. This asymmetrical weight-bearing affects the affected limb and can contribute to difficulties in maintaining balance and stability (4, 13). While Apriliyasari et al. (14) provide an excellent recent review of the effect of proprioceptive training in stroke rehabilitation, it is apparent that most of the interventions discussed do not target improving proprioception of the whole-body segments (13). Stroke patients may benefit from simulated axial loading that can be experienced by the sensory-motor system (6). Similar axial loading is also successfully used by astronauts who experience similar neuro-musculoskeletal changes in space, like that after a stroke.

### Similarities in physiological changes due to a stroke and exposure to microgravity

The human body in space experiences microgravity and adapts to a less demanding environment. As a result, astronauts often lack the strength, stamina, and cardiovascular endurance required to meet the demands of Earth’s gravitational forces immediately upon their return from spaceflight (13, 15). Astronauts come back with various physiological adaptations like muscle atrophy, gait disturbances, altered proprioceptive function, vestibular disturbances, cardiovascular deconditioning, fluid shifts to the upper body, loss of bone density, and low vision (15–21). These adaptations are observed more frequently during longer-duration spaceflight missions, spanning over weeks or months (6, 22–24).

These changes can resemble the physiological adaptations observed in patients who have undergone prolonged bed rest following a stroke (4, 6). In fact, researchers use bed-rest as one of the spaceflight analogs to force the inactivity of different parts of the human body, test out various countermeasures, and understand the physiological adaptations at the fundamental level (24). The inability to move independently and safely quickly leads to muscle atrophy, loss of strength, and cardiovascular deconditioning in stroke patients (25). The affected side post-stroke can also lead to decreased bone density and compromised bone integrity (4). Alteration of the sensorimotor and vestibular system can make it challenging to maintain an orthostatic position (26). There is also a migration of fluids from the lower extremities to the chest and head, increasing intracranial pressure, which can increase dizziness, lack of balance and low vision, making the patient prone to falls (26).

These similarities between astronauts and stroke patients, who both have experienced a period of limited mobility or/and bed rest, have increased the medical community’s interest in seeking alternative and effective rehabilitation techniques (24). Space missions have significantly contributed to our understanding of rehabilitation programs designed to counteract the detrimental physiological effects of reduced gravitational activation (6, 18, 27). Astronauts undergo intensive rehabilitation interventions to accelerate recovery (15). They also employ various exercise techniques or other countermeasures, like body suits while in space, to reduce the debilitating physiological effects on their bodies.

### Countermeasure loading suits for astronauts

To mitigate the harmful somatosensory and musculoskeletal deficits, the “Pingvin” (also sometimes called “Penguin”) suit was developed by Soviet scientists to be used by their cosmonauts during spaceflight (28). The suit featured an intricate system of elastic bands anchored at the waistband, generating vertical tension between the shoulders and waist and horizontally between the waist and feet. This axial loading challenged the cosmonaut’s muscles and core stability. While the suit was routinely used during Soviet spaceflights in the 1970s, 1980s, it was prone to overheating and discomfort and thus replaced by newer variants, like the Penguin-3 suit (29).

NASA has been funding the development of in-flight countermeasure suits for many years; for example, the Gravity Loading Countermeasure Suit (or the MIT Skinsuit) (30, 31) is a passive axial loading suit concept, but so far, we have only seen some pilot data from parabolic flights about its effectiveness or from a handful of spaceflights where it was used more as a technology demonstration. Similarly, a Torso Compression Harness to prevent spinal deconditioning (32) and a Variable Vector Countermeasure Suit (or V2 suit) (33) to provide “viscous resistance” against a selected “down” direction during movements are proposed and supported to some extent by NASA for initial feasibility. However, we have not seen quantitative data from experimental tests, especially in microgravity. An ESA-funded study proposed the concept of the ‘Dynasuit’ (34) in 2012 as an active, intelligent suit with bio-sensors and actuators in a biofeedback loop that included movement resistance and foot sole stimulation. However, we have yet to see this idea realized in the public domain.

## The TheraSuit Method®

### History of the TheraSuit Method®

Inspired by the early success of axial loading suits such as the Pingvin suit during spaceflight and recognizing that children with conditions like Cerebral Palsy (CP) experience similar movement difficulties as astronauts, the Pediatric Institute of the Russian Academy of Sciences developed and modified the Pingvin suit into various versions of the Adeli suit (e.g., Adeli-92, Adeli-94). These modified suits have been utilized to treat children with CP and other neuromuscular disorders. In 2000, a trained Polish physical therapist couple, Richard and Izabela Koscielny, whose daughter has Cerebral Palsy (CP), explored various rehabilitation strategies for her. They developed a variant of the Russian Adeli suit known as the TheraSuit® garment, along with the use of Universal Exercise Unit (UEU) and an intensive training program. This marked the first implementation of such a program in the United States, and they were awarded a patent for their invention under the name “TheraSuit.” The TheraSuit® garment is made of a soft dynamic orthosis, capable of providing significant sensory input of proprioception to increase functional movement in synergy (35). The development of the TheraSuit® garment is the facilitation of functional movement in all planes: sagittal, frontal, and transverse. The Therasuit Method® (that combines the garment, the UEU, and the intensive program) can be tailored to the individual needs of each patient with a neurological disorder. It is based on training principles for strength gains, respecting the peak physiological response of each patient. Care plans can be designed depending on the type of energy production, muscle fibers, and level of functioning. While there are other suits in the market in the US, like the Pediasuit, the Adeli suit, and the Neurosuit, to the best of our knowledge, TheraSuit® is the only method FDA certified in the United States. It has been used actively in various clinics around the world, mainly as part of a rehabilitation program for children with CP. While there are a few clinical trials done with the pediatric population with CP, and they have been successful in increasing gross motor skills, gait quality, and body alignment (36, 37), The TheraSuit Method® has not yet been applied in adult populations, particularly in the context of stroke rehabilitation; however, its principles hold potential for enhancing post-stroke rehabilitation outcomes.

### Pillars of the TheraSuit Method®

The TheraSuit Method® has several pillars that define its principles. This technique is not limited to TheraSuit® garment use only; it is based on intensive therapies using strength training principles and task-specific exercises involving repetitive movements and work against gravity employing the UEU.

#### Preparation

The body’s preparation includes hot packs and massages using neurological mobilization, traction, compressions, and vibration. This helps bring body awareness while stimulating sensory receptors in the skin, muscles, and joints (38).

##### Heat

The heat promotes vasodilation, helps eliminate lactic acid, and increases muscle oxygen (39). It also relaxes muscles and trigger points and decreases muscle tone (40).

##### Massage

Increases circulation and improve oxygenation, promoting muscle relaxation (40). The friction of the massage stimulates sensory receptors, relaxing painful muscles, and increasing blood flow (40). The massage also helps to break down connective tissue and promotes myofascial release.

##### Mobilization

Joint compressions, traction, and mobilization can be done to prepare the limb for resistive exercises in the pulley system. Mobilizations can shorten and elongate muscles, exposing nerves to tension and stretches and increasing the range of motion to facilitate exercises. Ranging and mobilizing the limbs can improve mobility and neuromuscular function (38).

#### Universal exercise unit

The Universal Exercise Unit (UEU) or “Cage” is heavy equipment made from metal with a grid wall designed and open in the front. It has a cubic or box shape measuring two meters by two meters by two meters. This equipment uses a system of cables, pulleys, weights, harnesses, and bungees. It can be used in various ways, such as pulleys for resistive exercises or the “spider” system for body suspension.

##### The Pulley system

**Figure 1:**
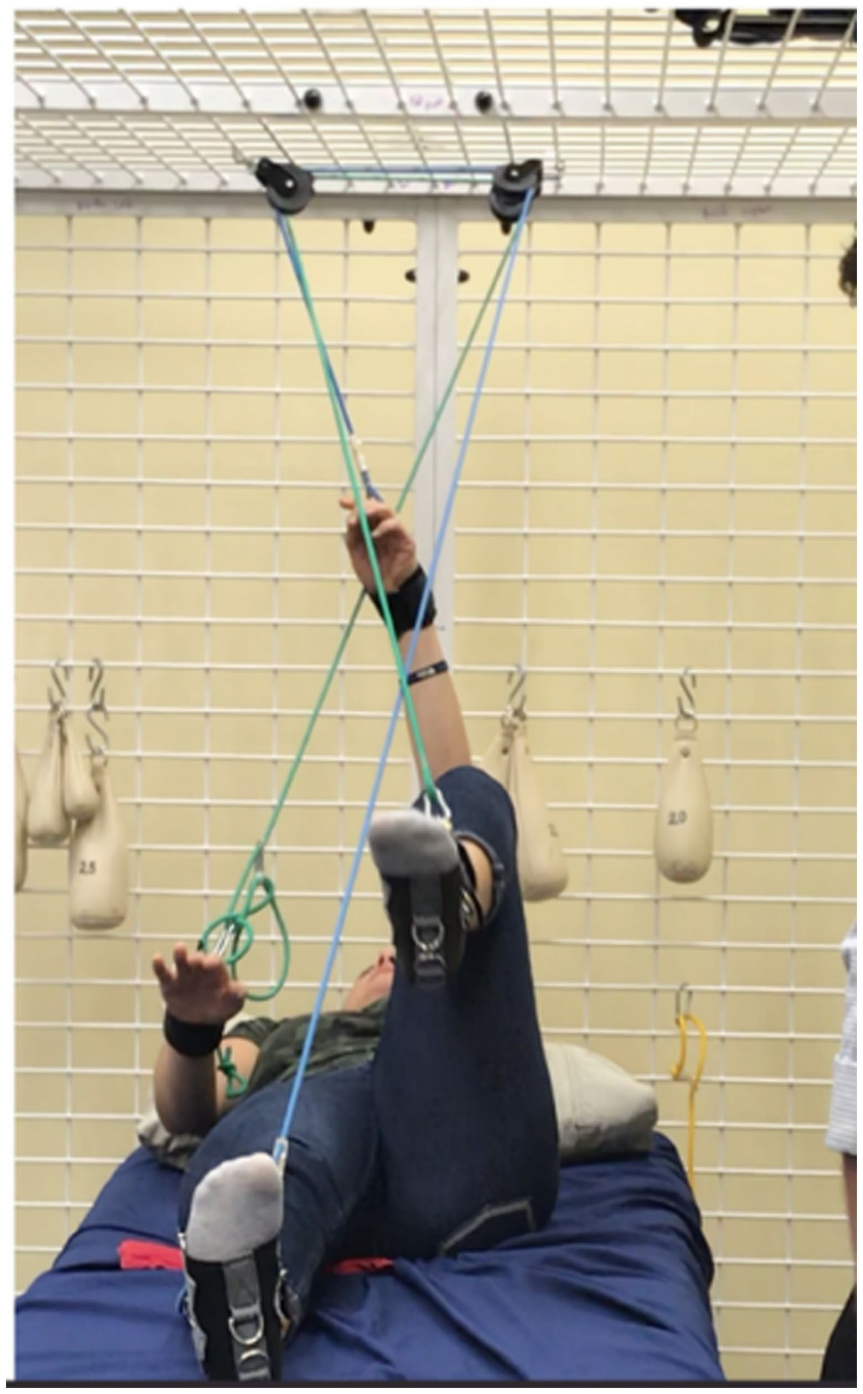
Pulley system.

The pulley allows the limbs to be suspended in gravity elimination to facilitate movement and help with complex movement patterns. This equipment utilizes weights to create resistance or facilitate movement. The goal is for the movement to be done actively. While working in the pulley system, sandbags are specially prepared to be used as an extra set of hands; they help hold a limb down or align the body. The UEU allows for the isolation of muscle groups and targets specific movements. Strength training promotes increased function, improving the quality of gait, balance, and coordination. Figure 1 is an example of the use of the pulley system.

##### The “Spider” suspension

**Figure 2:**
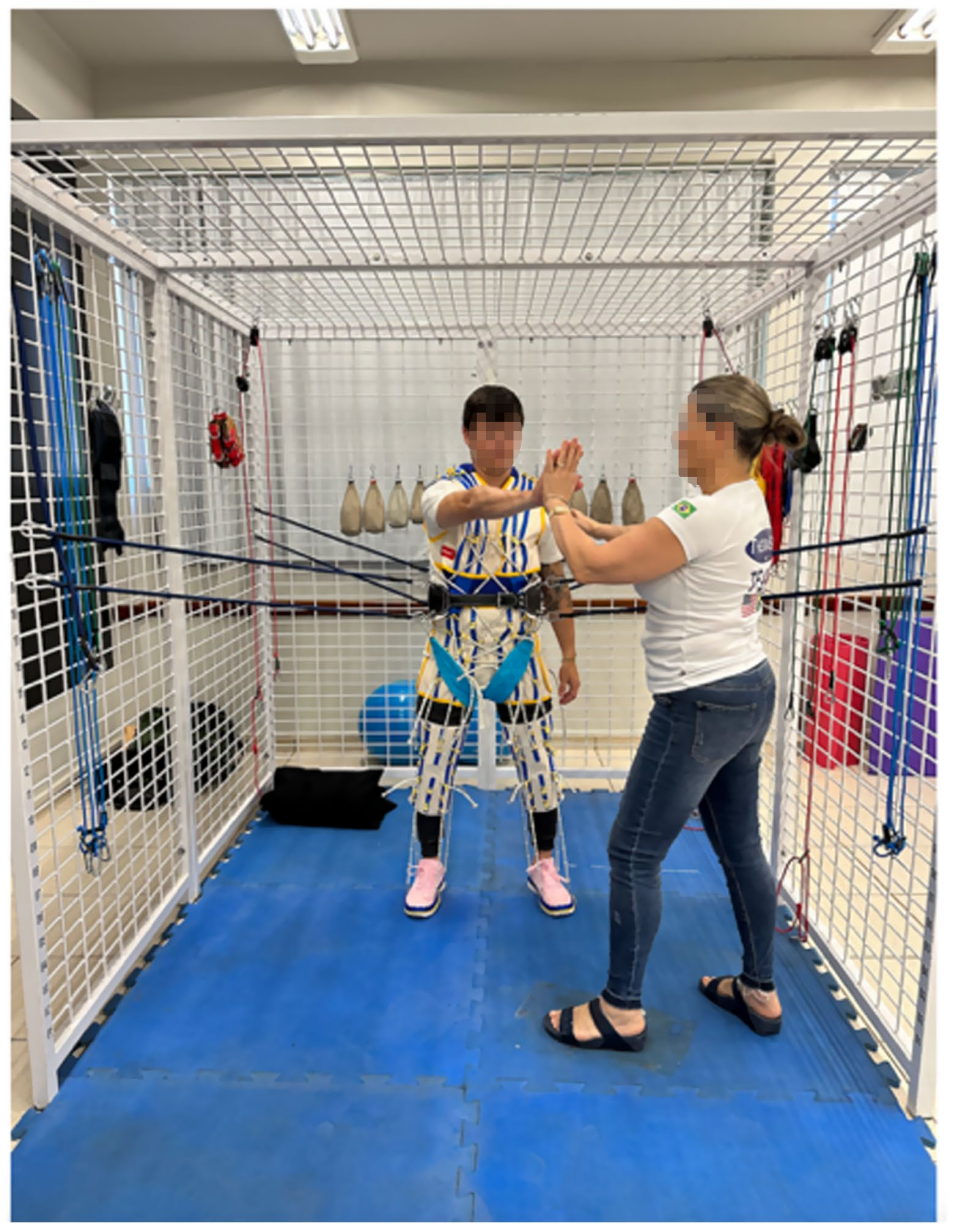
Example of exercise in the Universal Exercise Unit spider system.

The “spider” system allows the patient to be suspended by a belt connected to four or more bungees that attach to the cage, eliminating gravity and facilitating the standing position (41, 42). Bungees can also be attached lower on the cage and add weight and resistance to the moment in standing. The “spider” system supports safely shifting body weight, jumping, kneeling, half-kneeling, and standing. The “spider” cage is an effective tool to be able to stand a patient who is having balance and strength difficulties. Figure 2 shows an example of exercises in the UEU spider system.

#### TheraSuit® garment

The TheraSuit® garment is a soft, dynamic orthotic consisting of a hat, vest, shorts, knee pads, and shoes with rubber bands that attach to specifically placed hooks on the body in order to inhibit or facilitate movements (42). These rubber bands provide support through facilitation, resistance, inhibition, postural correction, and body alignment. It also offers dynamic feedback and deep proprioceptive input to the muscles, joints, and nervous system (36, 37). The TheraSuit® garment stabilizes the trunk and corrects posture, thus facilitating specific strength and functional tasks (35, 36).

The garment used, and the intensive intervention of resistive exercises represent the TheraSuit Method® (41). It is important to emphasize that the TheraSuit Method® is composed of pillars that include several components to promote strength and functional gains. These pillars include the preparation of the body to be worked on with massage, heat, vibration, and mobilization. This is followed by resistive exercises on the pulley system, facilitating activation against gravity. The following action is fitting the TheraSuit® garment to work on motor function on the floor, gait training, and other tasks. The treatment concludes with vestibular training and strengthening performed on the spider or pulley.

The TheraSuit® garment is one of the components of the TheraSuit Method®. It is used when there is a need to align the body, correct the posture, stabilize the trunk, and provide strong proprioceptive input (36, 37). In some instances, the TheraSuit® garment will not be used when applying the TheraSuit® Method®. The use of the TheraSuit® garment is contra-indicated in the presence of scoliosis, progressive metabolic disorders, joint degenerations, severe osteoporosis, severe subluxation, and fixed contractures (41, 42).

### Benefits of the TheraSuit Method®

Participation in more intensive therapies and resistive exercises offers benefits that may maintain strength (36, 37, 41). The use of the TheraSuit® garment is meant to increase the loading and proprioception in the joints and muscles of the affected side of the body. The elastic components used in the TheraSuit® garment provide resistance, compression, and axial loading to the body, generating stimulation of the Golgi tendon and creating a reflex response (43).

The TheraSuit Method® can retrain the central nervous system, helping to normalize muscle tone, correct gait pattern, improve balance, increase strength, and maintain bone density through impact and weight training (35, 36, 41, 42). Elastic bands can be used to correctly align the body, support weak muscles, and provide external stabilization (35, 36, 41, 42). The bands can be adjusted for each patient to provide support or resistance.

The TheraSuit Method® uses training principles with increased frequency and has demonstrated significant gains in gait patterns in children with CP (44, 45). Research studies have shown measurable strength gains by the third week of intensive treatment (36, 37). However, it is essential to emphasize that to maintain muscle strength, it is necessary to continue resistance exercises during intervals between one intervention and another (37).

Intensive therapy using the TheraSuit Method® has demonstrated results in increasing gross motor function to a greater extent than traditional therapies have shown promoting postutal correction (45–48). It has shown consistent improvement and positive effects on speed, cadence, and step length (44) In conclusion, despite the limited number of studies, there is evidence of this method’s effectiveness in treating children with neurological disorders.

## Discussion

Astronauts have been using countermeasures for many decades to reduce the physiological changes during spaceflight. One such focused countermeasure includes the stimulation of proprioception through axial loading suits in combination with strength training (15). The TheraSuit Method® has the potential to offer significant advantages in the recovery of patients with neurological disorders, such as stroke, here on Earth. This approach may enhance strength, increase resistance, improve balance, and overall patient functionality. Furthermore, the proprioceptive input delivered through this method can lead to notable improvements in trunk control, balance performance, gait, and mobility in post-stroke patients. By integrating these benefits, the TheraSuit Method® could play a pivotal role in facilitating more effective rehabilitation outcomes (14).

There is a limited number of peer-reviewed studies on the TheraSuit Method®, all focused on children (45, 48–50). To date, no studies have been conducted on the use of the TheraSuit Method® specifically within the context of post-stroke rehabilitation. This presents a significant gap in the literature, as understanding the application of the TheraSuit Method® in this area could offer valuable insights into its potential benefits.

It is crucial to explore these strengthening programs, as they have the potential to enhance functional gains for patients recovering from strokes. By investigating how the TheraSuit Method® can facilitate improvements in muscle strength, mobility, and overall functional ability, we may uncover effective strategies for rehabilitation that can significantly impact patient outcomes.

Conducting research in this domain will not only contribute to the evidence base for the TheraSuit Method® but also aid clinicians in developing comprehensive rehabilitation plans tailored to the unique needs of post-stroke patients. Ultimately, future research could lead to improved recovery trajectories and quality of life for individuals affected by stroke.
